# Higher Insulin Resistance and Adiposity in Postmenopausal Women With Breast Cancer Treated With Aromatase Inhibitors

**DOI:** 10.1210/jc.2018-02339

**Published:** 2019-03-28

**Authors:** Fraser W Gibb, J Michael Dixon, Catriona Clarke, Natalie Z Homer, Abdullah M M Faqehi, Ruth Andrew, Brian R Walker

**Affiliations:** 1 British Heart Foundation Centre for Cardiovascular Science, University of Edinburgh, Queen’s Medical Research Institute, Edinburgh, United Kingdom; 2 Edinburgh Breast Unit, Western General Hospital, Edinburgh, United Kingdom; 3 Clinical Biochemistry, Western General Hospital, Edinburgh, United Kingdom; 4 Institute of Genetic Medicine, Newcastle University, Newcastle Upon Tyne, United Kingdom

## Abstract

**Context:**

Aromatase deficiency causes obesity and insulin resistance in aromatase knockout mice and humans with rare mutations of the aromatase gene (*CYP19*). Aromatase inhibitors are a commonly prescribed therapy for postmenopausal breast cancer.

**Objective:**

We hypothesized that aromatase inhibitors induce obesity and insulin resistance when used in treatment of breast cancer.

**Design:**

Case-control study.

**Setting:**

University teaching hospital.

**Participants:**

Patients with postmenopausal breast cancer (n = 20) treated with aromatase inhibitors and 20 age-matched control subjects.

**Main outcome measures:**

The primary outcome measure was insulin sensitivity index – Matsuda, derived from a 75-g oral glucose tolerance test. Body composition was assessed by dual energy x-ray absorptiometry and biopsy specimens of subcutaneous adipose tissue obtained for assessment of mRNA transcript levels. Data are reported as mean ± SEM (patients receiving inhibitors vs control group, respectively).

**Results:**

Aromatase inhibitor therapy was associated with significantly lower insulin sensitivity (5.15 ± 0.45 vs 6.80 ± 0.64; *P* = 0.041), higher peak insulin concentration after oral glucose tolerance test (693.4 ± 78.6 vs 527.6 ± 85.5 pmol/L; *P* = 0.035), greater percentage of body fat (38.4% ± 1.0% vs 34.6% ± 1.3%; *P* = 0.026), and higher plasma leptin concentration (23.5 ± 2.8 vs 15.5 ± 2.3 ng/mL; *P* = 0.035).

**Conclusion:**

Women who received aromatase inhibitors for postmenopausal breast cancer had greater percentage body fat and insulin resistance compared with control subjects with no history of breast cancer.

## Teaser

We demonstrate increased insulin resistance, adiposity, and plasma leptin in patients with breast cancer treated with aromatase inhibitors, compared with control subjects matched for age and body mass index.

Manipulation of estrogen receptor (ER) signaling has been a central component in the management of hormone-receptor–positive breast carcinoma in postmenopausal women for over two decades. More than 1.5 million prescriptions are issued annually for hormonal breast cancer therapies in England (1). Tamoxifen (an ER partial agonist) and third-generation aromatase inhibitors (*e.g.*, anastrozole, letrozole, exemestane) are associated with increased disease-free survival. However, aromatase inhibitor therapy has been associated with an increased risk of cardiovascular disease (2), when compared with tamoxifen therapy. The effects of aromatase inhibition on lipid profile have been widely investigated (3), with mixed results, and do not necessarily represent a class effect.

After menopause, the previously lower risk of cardiovascular disease in women increases to converge with that of men (4). Type 2 diabetes appears to develop in men at a lower body mass index (BMI) than women (5), potentially as a consequence of estrogen-related differences in body fat distribution (6). Hormone replacement therapy reduces the risk of diabetes, with a number needed to treat of 30 to prevent one case (7). Postmenopausal plasma estradiol levels are ≥50% lower than those observed in men. It follows that aromatase inhibition, by further lowering circulating estradiol levels, may result in an exaggerated postmenopausal phenotype. This may be particularly pronounced in adipose tissue, because aromatase inhibitors have an even greater suppressive effect on estradiol in breast tumor tissue than on circulating estradiol concentration in women with breast cancer (8).

Aromatase knockout mice (9, 10) and rare human examples of congenital aromatase deficiency (11) are associated with increased adiposity, hepatic steatosis, and insulin resistance. We have previously reported increased insulin resistance in healthy men after 6 weeks’ treatment with an aromatase inhibitor (12). However, the effects of aromatase inhibition on insulin sensitivity have not been assessed in postmenopausal women, the group most likely to be exposed to aromatase inhibitor therapy. We sought to investigate this potential association by performing a case-control study comparing postmenopausal women with breast cancer treated with an aromatase inhibitor with age-matched healthy volunteers.

## Methods

### Study design

This was a case-control study, comparing women with breast cancer who were receiving aromatase inhibitor therapy, with age-matched healthy control subjects. Twenty postmenopausal women were recruited from the Edinburgh Breast Cancer Clinic. The inclusion criteria were a diagnosis of ER-positive breast carcinoma and current aromatase inhibitor therapy (either anastrozole, letrozole, or exemestane) for at least 1 year. Twenty age-matched postmenopausal control subjects without breast cancer or aromatase inhibitor therapy were recruited from the South East Scotland Breast Cancer Screening service. All participants were white European. Exclusion criteria for both groups were metastatic breast carcinoma, important medical comorbidities, hormone replacement therapy, previous diagnosis of diabetes mellitus and recent (*i.e.*, within 3 months) therapy with glucocorticoids. Ethical approval was granted from the Lothian Research Ethics Committee and all participants provided written informed consent.

### Study protocol

Subjects attended the clinical research facility at 8:30 am after an overnight fast and were asked to abstain from alcohol, tobacco, and caffeine starting the evening before attendance. An oral glucose tolerance test (sampling at −30, −15, 0, +30, +60, +90, and +120 minutes, in reference to 75 g of anhydrous glucose) and basic anthropometric measurements (*i.e.*, weight, height, waist and hip circumferences) were taken. Systolic and diastolic blood pressures and pulse rate were measured after sitting for ≥10 minutes, using a 705IT automatic blood pressure monitor (OMRON Health care, Hoofddorp, Netherlands). At completion of the oral glucose tolerance test, a subcutaneous adipose needle biopsy was performed (13). On a separate day, within 2 months of the first visit, subjects attended the Western General Hospital for a dual energy x-ray absorptiometry scan (Discovery A, Hologic, Bedford MA). Estimated bone mineral content, fat mass, lean mass, and percentage fat were reported for five separate compartments: head, trunk, left arm, right arm, left leg, and right leg. The proportion of android fat was calculated as follows: (trunk fat + arm fat)/total body fat. The proportion of gynoid fat was calculated as follows: leg fat/total body fat (14). The fat distribution index was the ratio of trunk to leg fat (15). The Scottish Index of Multiple Deprivation score was used to assess the deprivation status of participants (based on 38 measures across 7 categories in 6505 geographical “data zones”) (16).

### Laboratory assays

Fasting lipid profile (including total cholesterol, triglyceride, low-density lipoprotein cholesterol and high-density lipoprotein cholesterol levels) and glucose levels were analyzed on the Vitros platform (Ortho-Clinical Diagnostics, High Wycombe, UK); insulin was analyzed using a chemiluminescent microparticle immunoassay (Architect 8K41, Abbott Laboratories, Wiesbaden, Germany) and adiponectin, resistin, leptin, MCP-1, and IL-8 were analyzed by multiplex immunoassay (Merck Millipore, Watford, UK). Plasma testosterone, androstenedione, estradiol, and estrone levels were quantified by liquid chromatography–tandem mass spectrometry, as described previously (13, 17). SHBG was analyzed by immunoassay on the Roche E411 analyzer (Roche Diagnostics, Burgess Hill, UK).

Adipose tissue was processed, RNA extracted, and after reverse transcription, quantitative PCR was performed to analyze the mRNA transcript levels of a panel of genes related to adipogenesis, steroid metabolism, and adipocytokines, normalized against the abundance of cyclophilin (12). Cyclophilin expression was not different between patients and control subjects (*P* = 0.35).

### Data analysis and statistical methods

Insulin sensitivity index – Matsuda (hereafter, Matsuda index) was calculated as previously described (18). All statistical analyses were carried out using SPSS Statistics for Windows, version 19.0, software (IBM, Armonk, NY). Data are presented as mean ± SEM (patient group vs control group, respectively) unless otherwise stated.

Comparisons between patients and control subjects were performed using independent-samples Student *t* tests when data were normally distributed. When data were not normally distributed, as determined by the Kolmogorov-Smirnov test, logarithmic transformation was performed and subsequently compared with Student *t* tests if a normal distribution was obtained or by independent-samples Mann-Whitney *U* tests if transformed data remained not normally distributed. Assessment of the influence of covariates was performed using analysis of covariance. Correlation between normally distributed variables was performed using the Pearson correlation coefficient, and the influence of potential confounders was assessed by multiple linear regression. Participant numbers were chosen to provide 80% power to detect a 50% difference in insulin sensitivity index (19). Statistical significance was accepted at *P* < 0.05.

## Results

### Subject characteristics

Anastrozole was the aromatase inhibitor used most prevalently (n = 12), with the remaining participants receiving letrozole (n = 6) and exemestane (n = 2). The mean duration of aromatase inhibitor therapy was 27.4 ± 2.8 months. Patients and control subjects were well matched with respect to age [61.4 ± 1.4 (range, 51 to 72) vs 59.4 ± 1.0 (range, 52 to 67) years; *P* = 0.259], BMI (27.1 ± 0.8 vs 26.6 ± 1.0 kg/m^2^; *P* = 0.68), and Scottish Index of Multiple Deprivation scores (4806 ± 355 vs 5013 ± 419; *P* = 0.71). Of the 20 patients, five had previously received systemic chemotherapy, although this was not associated with important differences in any of the parameters measured. Regular medication use was largely limited to levothyroxine replacement (seven patients, one control subject) and antihypertensive agents (four patients, one control subject). Four patients were receiving bendroflumethiazide, but this was not associated with any difference in indices of insulin sensitivity. There was no difference in fasting lipid profile (Table 1) or systolic blood pressure between patients and control subjects (139 ± 4 vs 131 ± 3 mm Hg; *P* = 0.140) but diastolic blood pressure was higher in patients receiving aromatase inhibitor treatment (82 ± 2 vs 75 ± 2 mm Hg; *P* < 0.05).

**Table 1.** Fasting Lipid Profile, and Sex Steroid and Adipokine Levels

	Patients (n = 20)	Control Subjects (n = 20)	*P* ^*a*^
Total cholesterol, mmol/L	6.0 (0.1)	5.7 (0.2)	NS
HDL cholesterol, mmol/L	1.6 (0.1)	1.7 (0.1)	NS
LDL cholesterol, mmol/L	3.8 (0.1)	3.5 (0.2)	NS
Triglycerides, mmol/L	1.1 (0.1)	1.0 (0.1)	NS
Cholesterol to HDL-C ratio	3.8 (0.2)	3.5 (0.2)	NS
Estradiol, pmol/L	18.7 (0.8)	35.5 (1.2)	<0.001
Estrone, pmol/L	16.2 (0.7)	26.9 (1.1)	<0.001
Testosterone, nmol/L	0.66 (0.03)	0.70 (0.02)	NS
Androstenedione, nmol/L	0.47 (0.02)	0.54 (0.03)	0.04
Free androgen index	1.06 (0.14)	0.91(0.11)	NS
SHBG, nmol/L	82 (8)	77 (9)	NS
Testosterone to estradiol ratio	38.6 (2.1)	19.4 (1.5)	<0.001
Androstenedione to estrone ratio	34.8 (2.2)	18.3 (1.2)	<0.001
Leptin, ng/mL	23.5 (2.8)	15.5 (2.3)	0.035^*b*^
Adiponectin, μg/mL	43.0 (5.9)	35.4 (3.7)	NS
IL-8, pg/mL	7.6 (1.1)	6.7 (0.6)	NS
MCP-1, pg/mL	292.5 (33.8)	268.3 (20.2)	NS
Resistin, pg/mL	22.2 (1.1)	22.9 (1.9)	NS

Data are reported as mean (SEM).

Abbreviations: HDL, high-density lipoprotein; HDL-C, high-density lipoprotein cholesterol; LDL, low-density lipoprotein; NS, not significant.

^a^ Data were compared by Student *t* tests or independent-samples Mann-Whitney *U* test (where data were not normally distributed even after log transformation).

^b^ Mann-Whitney *U* test.

### Sex steroid hormones

Plasma estradiol and estrone concentrations were, respectively, 47% (*P* < 0.001) and 40% (*P* < 0.001) lower in patients treated with aromatase inhibitors than in control subjects (Table 1). Testosterone concentration and free androgen index were not significantly different between groups, although androstenedione concentration was 13% lower (*P* = 0.04) in patients treated with aromatase inhibitors (Table 1). Estradiol concentration was significantly negatively correlated with insulin sensitivity index in patients (*R* = −0.497; *P* = 0.03) but not control subjects (*R* = −0.006; *P* = 0.982) (20). The association between estradiol and insulin sensitivity index was no longer significant after adjustment for percentage body fat in a multiple regression model (20). No other significant correlations were observed between sex steroid hormones and either insulin sensitivity or percentage body fat (Table 2).

**Table 2.** Correlations Between Sex Steroids and Body Fat/Insulin Sensitivity

	Testosterone		Androstenedione		Estrone		Estradiol		Free Androgen Index		SHBG		Testosterone to Estradiol Ratio		Androstenedione to Estrone Ratio	
	P	C	P	C	P	C	P	C	P	C	P	C	P	C	P	C
Body fat, %	0.266	0.061	−0.114	−0.196	0.384	−0.183	0.351	0.267	0.448^*a*^	0.552^*a*^	−0.504^*a*^	−0.687^*a*^	−0.156	−0.292	−0.365	−0.063
ISI	−0.265	−0.112	−0.062	−0.381	−0.340	0.196	−0.497^*a*^	−0.006	−0.391	−0.503^*a*^	0.467^*a*^	0.571^*a*^	0.312	−0.091	0.382	−0.303

Data are presented as Pearson *R* correlation coefficients.

Abbreviations: C, control subjects; ISI, insulin sensitivity index; P, patients.

^a^ *P* < 0.05.

### Body composition

Although there were no significant differences in anthropometric measures of body composition (Table 3), dual-energy x-ray absorptiometry revealed lower lean mass in patients than in control subjects across almost all compartments (Table 3). Total fat mass and trunk fat were not different, but peripheral percentage body fat was greater in patients receiving aromatase inhibitor treatments than in control subjects (Table 3). Fat distribution index (*i.e.*, the ratio of trunk to leg fat) did not differ between groups.

**Table 3.** Anthropometric Measurements and detailed Dual Energy X-Ray Absorptiometry Body Composition Analysis

	Patients (n = 20)	Control Subjects (n = 20)	*P* ^*a*^
Height, cm	159.8 (0.9)	165.1 (1.7)	0.001
Weight, kg	69.2 (2.3)	72.5 (2.9)	NS
BMI, kg/m^2^	27.1 (0.9)	26.6 (1.0)	NS
Waist circumference, cm	90.1 (2.0)	88.4 (2.6)	NS
Hip circumference, cm	102.3 (1.6)	103.4 (1.8)	NS
Waist to hip ratio	0.88 (0.01)	0.85 (0.01)	NS
Total bone mineral content, kg	1.92 (0.06)	2.05 (0.08)	NS
Total body fat, kg	26.72 (1.49)	25.61 (2.01)	NS
Total body lean mass, kg	40.28 (9.29)	44.72 (14.94)	0.014
Total body fat, %	38.4 (1.0)	34.6 (1.3)	0.026
Fat distribution index	1.3 (0.1)	1.3 (0.1)	NS
Proportion android fat	0.6 (0.01)	0.6 (0.02)	NS
Proportion gynoid fat	0.4 (0.01)	0.4 (0.01)	NS

Data presented as mean (SEM).

Abbreviation: NS, not significant.

^a^ Compared by Student *t* test.

### Insulin sensitivity

**Figure 1.**
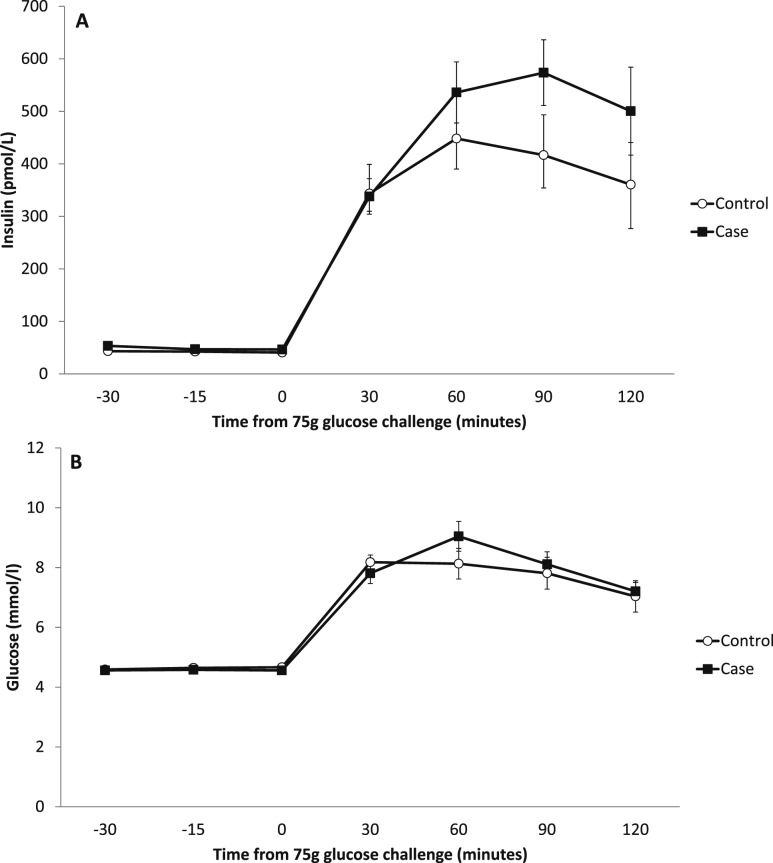
Graphs showing changes in (A) plasma insulin and (B) glucose levels across 2-hour 75-g oral glucose tolerance test. Data are reported as mean ± SEM.

All 40 participants had normal fasting blood glucose levels (*i.e.*, <6 mmol/L), with six participants in the aromatase inhibitor group having impaired glucose tolerance (glucose level, 7.8 to 11.0 mmol/L at 2 hours after ingesting 75 g of oral glucose) compared with three participants in the control group. In addition, two participants in the control group had 2-hour glucose levels in the diagnostic range for diabetes (*i.e.*, >11 mmol/L). Three patients and two control subjects fulfilled the International Diabetes Federation criteria for metabolic syndrome. Patients receiving aromatase inhibitor treatment were significantly more insulin resistant than control subjects, with a 24% lower Matsuda index (5.15 ± 0.45 vs 6.80 ± 0.64; *P* = 0.041), an effect independent of age but not body fat percentage. Peak insulin concentration was also greater in patients than in control subjects (693.4 ± 78.6 vs 527.6 ± 85.5 pmol/L; *P* = 0.035; Fig. 1). Homeostatic model assessment of insulin resistance (HOMA-IR) did not differ between patients and control subjects (1.41 ± 0.16 vs 1.23 ± 0.16; *P* = 0.365).

### Adipokines and proinflammatory cytokines

Serum leptin levels were higher in patients receiving aromatase inhibitors (Table 1), although this relationship did not persist when adjusted for percentage body fat. IL-8, MCP-1, adiponectin, and resistin values did not differ between patients and control subjects (Table 1).

### Subcutaneous adipose tissue mRNA

In subcutaneous adipose tissue, increased abundance of mRNA of genes encoding LKB1 (32%; *P* = 0.03), *β*-catenin (27%; *P* = 0.023), and leptin (40%; *P* = 0.003) was observed in patients. There were nonsignificant trends toward greater expression of perilipin 2 (21%; *P* = 0.077), PPAR*γ* (25%; *P* = 0.087), and lipoprotein lipase (14%; *P* = 0.086) in patients compared with control subjects. The full results for all genes assessed are presented in Table 4.

**Table 4.** Subcutaneous Adipose Tissue mRNA Transcript Levels in Patients Treated With Aromatase Inhibitors and in Control Subjects

	Patients (n = 20)	Control Subjects (n = 18)	*P*
Steroid hormone synthesis and metabolism			
* HSD11B1*, 11*β*HSD1	0.91 ± 0.15	0.78 ± 0.10	NS^*a*^
* AKR1C2*, aldo-keto reductase 1C2	1.01 ± 0.20	0.80 ± 0.11	NS^*a*^
* CYP19A1* aromatase	0.66 ± 0.14	0.42 ± 0.09	NS^*a*^
Steroid hormone receptors			
AR, androgen receptor	0.77 ± 0.06	0.71 ± 0.05	NS^*a*^
* ESR1*, estrogen receptor *α*	0.74 ± 0.10	0.59 ± 0.07	NS^*a*^
* ESR2*, estrogen receptor *β*	0.41 ± 0.03	0.42 ± 0.03	NS
Adipogenesis, lipogenesis, and lipolysis			
* ACACA*, acetyl CoA carboxylase	0.76 ± 0.09	0.65 ± 0.11	NS
* UCP2*, uncoupling protein 2	0.44 ± 0.04	0.46 ± 0.03	NS^*a*^
* FASN*, fatty acid synthase	0.65 ± 0.07	0.62 ± 0.07	NS
* LIPE*, hormone-sensitive lipase	1.28 ± 0.17	0.99 ± 0.14	NS
* PLIN2*, perilipin 2	0.70 ± 0.07	0.55 ± 0.05	NS
* LKB1*, liver kinase B1	1.44 ± 0.19	0.98 ± 0.15	0.030^*a*^
* CTNNB1*, *β*-catenin	0.62 ± 0.04	0.45 ± 0.02	0.023
* PNPLA2*, adipose triglyceride lipase	0.58 ± 0.07	0.62 ± 0.08	NS^*a*^
* PPARG*, peroxisome proliferator-activated receptor *γ*	0.91 ± 0.11	0.68 ± 0.10	NS^*a*^
* PPARGC1A*, PGC-1*α*	0.67 ± 0.08	0.58 ± 0.08	NS
Lipid and sterol metabolism			
* CETP*, cholesterol ester transfer protein	0.62 ± 0.14	0.90 ± 0.24	NS^*a*^
* LPL*, lipoprotein lipase	1.25 ± 0.07	1.08 ± 0.14	NS^*a*^
* SREBF1*, sterol regulatory element binding transcription factor 1	0.86 ± 0.10	0.70 ± 0.12	NS
* SREBF2*, sterol regulatory element binding transcription factor 2	0.76 ±	0.64 ±	NS^*a*^
Adipokines			
* IL6*, interleukin-6	0.87 ± 0.15	1.00 ± 0.20	NS^*a*^
* LEP*, leptin	0.92 ± 0.10	0.55 ± 0.06	0.003^*a*^
* ADIPOQ*, adiponectin	1.28 ± 0.10	1.17 ± 0.06	NS^*b*^
Miscellaneous			
* IGF1R*, IGF-1 receptor	0.73 ± 0.06	0.62 ± 0.04	NS
* IGF1*, insulin-like growth factor-1	0.67 ± 0.05	0.64 ± 0.05	NS
* AGT*, angiotensinogen	0.86 ± 0.08	0.95 ± 0.14	NS
* ADRA2A*, *α*-2-adrenergic receptor	0.95 ± 0.09	0.83 ± 0.11	NS^*a*^
* ARDB1*, *β*-1-adrenergic receptor	0.74 ± 0.07	0.65 ± 0.05	NS^*a*^

Data are presented as mean ± SEM (relative to cyclophyllin).

Abbreviation: NS, not significant.

^a^ Compared with independent-samples Student *t* test (data log transformed where not normally distributed as determined by Kolmogorov-Smirnov test).

^b^ Independent-samples Mann-Whitney *U* test where transformation did not result in normally distributed data.

## Discussion

These data show that women with breast cancer treated with aromatase inhibitors are more insulin resistant and have greater percentage body fat than healthy control subjects. The primary outcome measure was the Matsuda index, which was selected because it integrates information from the glucose and insulin values in the fasting and fed states to provide an estimate of insulin sensitivity that accords well with gold standard glucose clamp studies (18). We previously demonstrated decreased peripheral glucose disposal in healthy men treated with anastrozole (12), and the hyperinsulinemia after a glucose load demonstrated in the current study is consistent with a similar effect in postmenopausal women. We have not tested the underlying mechanism but suspect it relates to estrogen deficiency following aromatase inhibition, particularly because circulating estrogens were lower in women treated with an aromatase inhibitor, but androgens were not elevated. Our hypothesis is supported by emerging observational data suggesting a greater than fourfold increased risk of incident diabetes associated with aromatase inhibitor therapy in postmenopausal women with breast cancer (21). In the patients treated with aromatase inhibitors (but not control subjects), plasma estradiol level was negatively associated with insulin sensitivity. This was not independent of percentage body fat and is consistent with previous observations that aromatase inhibitor suppression of plasma estradiol is attenuated in obesity (22). Indeed, target-tissue estrogen levels correlate poorly with plasma levels (23), and most estrogen action in postmenopausal women is likely to be mediated by local generation at target tissues (*e.g.* 20-fold higher estradiol in fat than plasma) (24).

Estrogen deficiency at menopausal transition is associated with increased visceral adiposity and its attendant associations with cardiometabolic risk factors (25). Conceivably, by additional profound suppression of estrogen, aromatase inhibition has the potential to exacerbate this phenotype. Only two previous studies have assessed the effect of aromatase inhibition on body composition and both were potentially confounded by participants in the comparator groups receiving tamoxifen, which may increase visceral adiposity (26). In one study, 11 recently menopausal participants with breast cancer who were receiving aromatase inhibitors were compared with 71 women receiving alternative therapies (mostly tamoxifen). Over 24 months, those treated with aromatase inhibitors gained lean mass, whereas those not receiving aromatase inhibitors had increased body fat (27). Our findings of reduced lean mass and greater percentage body fat with correspondingly elevated serum leptin are not entirely congruent with these earlier investigations, but our comparator group did not receive tamoxifen. However, our findings of lower lean mass, higher serum leptin levels, and higher adipose tissue mRNA for lipoprotein lipase and leptin are consistent with estrogen deficiency and opposite to known estrogen effects on fat and muscle (28–30). Moreover, similar elevations in adipose leptin mRNA levels were observed in aromatase knockout mice, and the levels were lowered by estradiol administration (31).

We assessed a range of mRNA transcript levels in subcutaneous adipose tissue, on the basis of previous evidence of regulation of expression by sex hormones, but surprisingly, we did not find many changes. *β*-Catenin transcript levels, a central component of WNT signaling regulated by sex steroids (32, 33), were higher in women treated with aromatase inhibitors. LKB1, an upstream activator of AMPK upregulated by estradiol (34) that promotes fatty acid oxidation and suppresses fatty acid synthesis in adipocytes, had paradoxically higher transcript levels in women receiving aromatase inhibitor treatment, although we did not assess LKB1 phosphorylation. The differences in transcript levels were consistent with an antiadipogenic effect (increased LKB1 and *β*-catenin), even though fat mass was increased and leptin mRNA levels were higher with aromatase inhibition.

In this study, diastolic (but not systolic) blood pressure was higher in patients receiving aromatase inhibitor treatment than in control subjects, despite a higher proportion of antihypertensive therapy in the former. Polymorphisms in *CYP19* have been associated with differences in diastolic blood pressure in women (35). The lack of difference in fasting lipid profile with aromatase inhibition is broadly in accord with existing evidence. Prospective evaluation of cholesterol after anastrozole therapy did not detect any significant difference in fasting lipid profile (36).

The major limitation of the study is the possibility of confounding by the history of breast cancer only among the group taking aromatase inhibitors. We achieved satisfactory matching with respect to age, weight, waist circumference, and BMI between groups. The BMI matching addressed a potentially important limitation of the case-control design, because increasing BMI is associated with a higher risk of breast cancer in postmenopausal women (37). Diabetes (38), metabolic syndrome (39), and insulin resistance (40) have been reported as risk factors for breast cancer, although the relationship with diabetes is attenuated by correction for BMI (41). Furthermore, our cohort was well matched for glucose tolerance status and no differences were observed in fasting insulin resistance as determined by HOMA-IR. This is consistent with our previous observation that aromatase inhibition is likely to mediate increased insulin resistance through reduced peripheral glucose disposal in skeletal muscle (12) (captured by the dynamic insulin sensitivity index but not the static HOMA-IR). Although there is a recognized association between increased height and postmenopausal breast cancer risk (37), the mean height of the women with breast cancer was shorter than that of the control group in this study. Attendance at breast cancer screening (control group) is associated with affluence (42), which, in turn, is associated with lower prevalence of obesity and type 2 diabetes (43); however, we found no evidence of disparity in affluence between groups. With the exception of breast cancer, patients and control subjects were generally in good health, as mandated by the study exclusion criteria. There was a disparity in levothyroxine-treated participants between groups. If levothyroxine therapy is associated with alterations in body composition or insulin sensitivity, this could represent a confounder. Reassuringly, postmenopausal women treated with thyroidectomy and subsequent levothyroxine did not change in weight or body composition (44). Thiazide diuretic use has been associated with altered glucose homeostasis (through attenuated insulin secretion) (45), although a large prospective study found no increased diabetes risk with thiazide diuretics (46). Overall, therefore, we did not find evidence to support confounding of our primary observations by selection bias operating between the two groups.

In conclusion, as hypothesized, aromatase inhibition was associated with insulin resistance and differences in body composition, although this manifested as reduced lean mass rather than, as predicted, a shift from peripheral to central adiposity. These results have potentially important clinical implications and should prompt a more comprehensive assessment of the metabolic effects of aromatase inhibitors in women with breast cancer.

## Abbreviations:

BMI: body mass index
ER: estrogen receptor
HOMA-IR: homeostatic model assessment of insulin resistance
